# Anodal transcranial direct current stimulation over the left dorsolateral prefrontal cortex modulates attention and pain in fibromyalgia: randomized clinical trial

**DOI:** 10.1038/s41598-017-00185-w

**Published:** 2017-03-09

**Authors:** Adriana Ferreira Silva, Maxciel Zortea, Sandra Carvalho, Jorge Leite, Iraci Lucena da Silva Torres, Felipe Fregni, Wolnei Caumo

**Affiliations:** 1 Post Graduate Program in Medical Sciences, School of Medicine, Federal University of Rio Grande do Sul, Porto Alegre, Brazil; 2 Laboratory of Pain & Neuromodulation, Clinical Hospital of Porto Alegre, Porto Alegre, Brazil; 3 Spaulding Center of Neuromodulation, Department of Physical Medicine and Rehabilitation, Harvard Medical School, Boston, Massachusetts USA; 4 Neuropsychophysiology Laboratory, CIPsi, School of Psychology (EPsi), University of Minho, Braga, Portugal; 5 Pharmacology Department, Institute of Basic Health Sciences, Federal University of Rio Grande do Sul, Porto Alegre, Brazil; 6 Pain and Palliative Care Service, Clinical Hospital of Porto Alegre, Porto Alegre, Brazil; 7 Surgery Department, School of Medicine, Federal University of Rio Grande do Sul, Porto Alegre, Brazil

## Abstract

Cognitive dysfunction in fibromyalgia patients has been reported, especially when increased attentional demands are required. Transcranial direct current stimulation (tDCS) over the dorsolateral prefrontal cortex (DLPFC) has been effective in modulating attention. We tested the effects of a single session of tDCS coupled with a Go/No-go task in modulating three distinct attentional networks: alertness, orienting and executive control. Secondarily, the effect on pain measures was evaluated. Forty females with fibromyalgia were randomized to receive active or sham tDCS. Anodal stimulation (1 mA, 20 min) was applied over the DLPFC. Attention indices were assessed using the Attention Network Test (ANT). Heat pain threshold (HPTh) and tolerance (HPTo) were measured. Active compared to sham tDCS led to increased performance in the orienting (mean difference [MD] = 14.63) and executive (MD = 21.00) attention networks. There was no effect on alertness. Active tDCS increased HPTh as compared to sham (MD = 1.93) and HPTo (MD = 1.52). Regression analysis showed the effect on executive attention is mostly independent of the effect on pain. DLPFC may be an important target for neurostimulation therapies in addition to the primary motor cortex for patients who do not respond adequately to neurostimulation therapies.

## Introduction

Fibromyalgia (FM) is a chronic and widespread musculoskeletal pain disorder that commonly manifests itself as body stiffness, fatigue, sleep disorders, anxiety, depression, with a highly subjective pain catastrophizing^1^. An important mechanism involved in its physiopathology is an excessive cortical facilitation (a lack of inhibition)^2^, which has been associated with a lower pain threshold, and a higher level of trait anxiety^3, 4^. It is thought that FM is related to abnormal information across the afferent pathways to the brain that, due to the phenomenon of central sensitization, changes the brain sensory processing, which in turn leads to the chronic sensation of pain. This process involves regions defined as the pain neuromatrix, which consists of the frontal lobe, anterior cingulate cortex (ACC), insula, amygdala, hypothalamus, periaqueductal gray (PAG), nucleus cuneiformis (NCF), and rostral ventromedial medulla (RVM)^5^.

In addition to pain, cognitive impairments are reported in more than 70% of subjects with FM^6, 7^. Overall, subjects with FM show deficits in tasks requiring working memory, attention, conflict monitoring and verbal fluency^8^. Despite the fact that there is mixed evidence about attentional impairments in patients with FM^9^, several studies have demonstrated that subjects with FM have impaired attention in tasks involving competing information, reduced vigilance as showed by slower reaction time; and greater alertness as showed by greater reduction in errors after warning cues^8, 10^. Moreover, these attention deficits have been associated with other clinical symptoms such as sleep quality, anxiety and depression levels, and pain catastrophizing^8^.

The attention network model^11^ separates it into three components: Alerting is the achievement and maintenance of an alert state. Orienting refers to the selection and use of a stimulus from the sensory system. Finally, Executive control of attention deals with conflicting information to give a response. This relative independent component of attention can be measured separately using the Attention Network Test (ANT)^12^. Alerting attention is supposed to be processed by the thalamus and involves activation of anterior and posterior cortical sites. Orienting attention tends to activate superior parietal locations and pre-central gyrus, which is closely related to frontal eye fields. Anterior cingulate and other cortical left and right frontal areas are activated when Executive control of attention is performed^13^. This anatomo-clinical relationship creates an opportunity to investigate the effect of non-invasive brain stimulation techniques, such as transcranial direct current stimulation (tDCS).

tDCS is a safe, painless and effective non-invasive brain stimulation technique that is able to neuromodulate cortical areas by decreasing (anodal stimulation) or increasing (cathodal stimulation) neuronal firing threshold^14^. Indeed, in healthy subjects tDCS over the dorsolateral prefrontal cortex (DLPFC) increased pain threshold^15^ and in patients with fibromyalgia it relieved chronic pain^16, 17^. This technique has been effectively used to enhance cognitive functions, in both healthy^18–20^ and clinical populations^21, 22^. Regarding cognitive function, recent findings suggest that the effects of tDCS can be boosted in association with a task^23^. The underlying mechanism is the neuronal long-term potentiation (LTP), in which a strong synaptic stimulation can lead to a strengthening of the synaptic transmission.

The DLPFC is an important brain region for emotional processing and down regulation of affective conditions such as pain and plays an important role in several cognitive processes, such as cognitive flexibility, working memory, and planning^24^. To enhance the effects of tDCS over the DLPFC, a combined task should recruit the functioning of this area. The Go/No-go Task is known for requiring attention and the inhibition of a response when certain conditions are presented. Moreover, the performance on this task is related to the functioning of prefrontal areas, including the DLPFC^25^. Even though most of FM patients show attentional impairments, little is known about the effects of attentional processing modulation on pain perception in these patients. Nevertheless, there is some evidence from healthy subjects that top-down attentional processes are involved in nociceptive modulation^26^.

Here, we investigate the effects of tDCS over the DLPFC coupled with a Go/No-go Task in modulating three distinct attentional networks: alert, orienting and executive control. We hypothesize that the active (a)-tDCS, as compared to sham, will lead to significant changes in attention performance. In addition, the neuromodulation of one component of the pain neuromatrix will also lead to changes in pain measurements.

## Results

### Patient characteristics

The clinical and demographic characteristics of the subjects according to the sequence allocation were comparable and are shown in Table 1. Twenty patients were allocated according to the sequence Sham-Active Group, and twenty were assigned to the sequence Active-Sham Group. Two subjects were excluded because they did not reliably understand the experiment procedures and instructions and two other subjects were excluded due to extreme internal variability in pain measures and the ANT task. In the group receiving sham first, one subject dropped out without a justification reported. Thirty-five subjects completed the study. Minor side effects (i.e. tingling, burning and itching) were presented by 33.33% (13/39) of subjects in the s-tDCS condition and 27.5% (11/40) in the a-tDCS condition. No major side effects were observed, and only 13.2% of subjects were able to correctly guess the intervention received.

**Table 1:** Sample Characteristics at Baseline, According to Group and Phase. Values are given as mean (SD) or frequency (%) (n = 40).

Variable	s-tDCS + Go/No-Go first (n = 20)	a-tDCS + Go/No-Go first (n = 20)	P value^b^
Age (years)	51.3 (9.2)	48.7 (9.9)	0.53
Body index weight (kg)	28.4 (4.3)	29.6 (7.4)	0.55
Education (years)	8.8 (3.7)	10.7 (4.0)	0.08
Smoking (yes)	5 (20%)	3 (15%)	0.31
Alcohol consumption (yes)	5 (25%)	1 (5%)	0.08
Clinical comorbidity	10 (50%)	8 (40%)	0.52
Diagnosis for psychiatric disorder (yes)^a^	12 (60%)	14 (70%)	0.45
Scores on BDI-II	25.0 (7.0)	24.0 (8.3)	0.61
Scores on VAS (0 to100) (cm)	7.31 (2.4)	7.7 (2.1)	0.82
Fibromyalgia Impact Questionnaire	64.8 (12.8)	66.8 (13.8)	0.80
Pittsburgh Sleep Questionnaire	13.3 (4.3)	11.9 (4.7)	0.32
Pain catastrophizing Scale	30.7 (11.8)	32.2 (14.6)	0.66
STAI - State	29.9 (7.8)	27.3 (8.4)	0.31
STAI - Trait	27.9 (5.4)	27.0 (7.2)	0.68
HPTh (°C)	40.8 (2.9)	39.9 (3.2)	0.31
HPTo (°C)	45.5 (2.8)	45.6 (3.1)	0.96
Central nervous system active medication (yes)	13 (65%)	13 (65%)	0.68
Antidepressant (yes)	10	8	—
Anticonvulsant (yes)	3	1	—

Notes: BDI-II: Back Depression Inventory II; VAS: visual analog scale; STAI: State Trait Anxiety Inventory.

^a^Based on the Structured Clinical Interview for DSM-IV Axis I Disorders (SCID-I). Patients could have none or more than one psychiatric disorder.

^b^Independent samples t-Tests for mean values and Chi-Square or Fisher’s tests for frequency values.

### Primary outcome: anodal tDCS over left DLPFC effects on ANT measures

**Figure 1:**
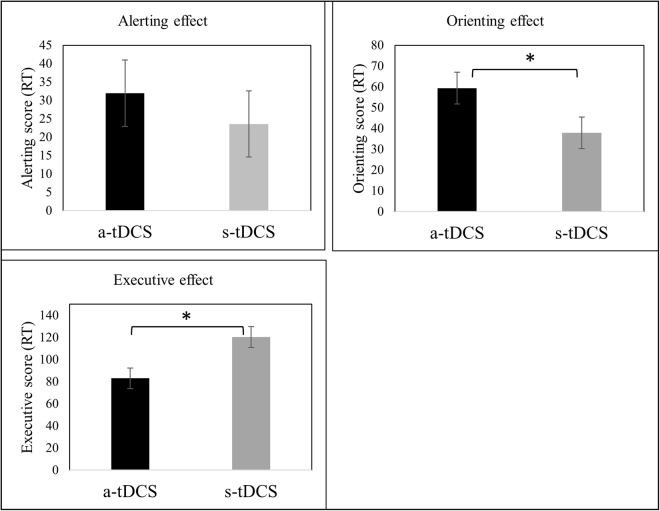
Group differences (main effects) on the ANT scores based on mixed linear model analyses. A: Alerting. B: Orienting. C: Executive. *P < 0,05.

We first analyzed Alerting, Orienting and Executive Attention indices using linear mixed models, considering two factors: Group (a-tDCS and s-tDCS) and Phase (first and second). For the Alerting index, no significant differences between Group, F(1, 66.24) = 0.953; P = 0.33, and Phase, F(1, 66.24) = 0.696; P = 0.40, were found, and no interaction was found, F(1, 66.24) = 0.989; P = 0.324 (Fig. 1A). For the Orienting index, there was a significant effect of Group, F(1, 70.0) = 4.189; P = 0.044, and no Phase effect, F(1, 69.18) = 0.145; P = 0.705. Although the interaction was not significant, F(1, 69.18) = 1.138; P = 0.290, groups differed only after the second crossover phase (p = 0.032), favoring the active group (M = 62.5; SE = 10.1) compared to sham group (M = 31.2; SE = 9.6), considering a Least Significant Difference (LSD) posthoc pairwise comparison (Fig. 1B). Finally, the Executive index showed a main effect for Group, F(1, 49.11) = 7.94; p = 0.007, and no effect of Phase, F(1, 50.53) = 0.014; p = 0.907. Although no significant interaction was found, *F*(1, 50.53) = 0.005; p = 0.946, a pairwise comparison (LSD posthoc) showed that only for Phase 2 (p = 0.013) the active tDCS group had less influence of the incongruent target (M = 83.2; SE = 10,2) than the sham group (M = 120.8; SE = 10,2, Fig. 1C). Table 2 presents group comparisons for the ANT scores differences between phases (first and second).

**Table 2:** Reaction Times (RTs) For Each Stimulation Group According to Target Type and Cue Type (n = 35).

Target Type	Cue Type	s-tDCS	a-tDCS	P value^a^
		M (SE)	M (SE)	
Congruent target	No cue	694.54 (19.2)	637.65 (19.2)	0.040
	Center	672.96 (22.8)	614.14 (22.8)	0.072
	Double	651.5 (20.8)	594.12 (20.8)	0.055
	Spatial	658.22 (23.5)	579.2 (23.5)	0.020
Incongruent target	No cue	789.1 (21.7)	736.5 (21.7)	0.091
	Center	768.7 (22.7)	742.4 (22.7)	0.415
	Double	740.9 (24.3)	731.3 (24.3)	0.780
	Spatial	709.3 (23.2)	691.3 (23.2)	0.584

Notes: M = mean; SE = standard error.

^a^Main effects based on mixed linear model analyses.

Additionally, we ran mixed linear model analyses for each of the Cues vs. Target conditions (except for neutral targets, due to its similarity with the congruent target conditions^12^), considering only main effects for Group, which is the factor of most interest. The a-tDCS group showed significantly better performance than the s-tDCS group in the congruent target conditions when there was no cue and spatial cue. Table 3 presents this data.

**Table 3:** Means and Standard Errors (SE) for the Primary Outcomes (scores of Alerting, Orienting and Executive Attention in the ANT) according to Group (a-tDCS + Go/no-Go Task *vs.* s-tDCS + Go/no-Go Task) (n = 35).

ANT scores	Sham tDCS M (SE)	Active tDCS M (SE)	Mean Difference (95% Confidence interval)	P value^a^
Alerting	8.98 (29.53)	12.16 (32.30)	3.17 (−4.88 to 11.22)	0.40
Orienting	39.36 (39.51)	53.99 (38.80)	14.63 (0.37 to 18.89)	0.04
Executive	122.98 (36.49)	88.68 (29.25)	−21.00 (−37.89 to − 4.11)	0.01

^a^Values based on a mixed ANOVA model. Significance level was P < 0.05.

### Secondary outcome: anodal tDCS over left DLPFC effects on HPTh and HPTo

Using a mixed linear model analysis to compare the Group and Phase, we observed a significant effect of Group related to HPTh, F (1; 11.06) = 24.85, P < 0.001, and a significant main effect of Phase, F (3; 11.06) = 6.92, P < 0.001. Also, there was an interaction between Group and Phase, F (3; 11.06) = 15.18, P < 0.001. Anodal tDCS over the left DLPFC was superior to sham on HPTh increase (irrespective of the Phase). HPTh improved 4.95% after anodal tDCS (the mean increase compared to the sham period), considering a pairwise comparison (LSD posthoc).

Also, a mixed model analysis revealed a significant effect of Group on the HPTo, F (1; 51.32) = 3.96, P = 0.03 and a significant main effect of Phase, F (3; 51.32) = 8.92, P = 0.01. Also, there was an interaction between Group and Phase, F (3; 51.32) = 3.34, P = 0.02. HPTo improved 3.6% during sustained heat pain stimulus (Table 4) after anodal tDCS (the mean increase compared to the sham period), considering a pairwise comparison (LSD posthoc), irrespective of the Phase. The effect of active tDCS compared to sham determined a large size effect on HPTh (0.89) and moderate size effect on HPTo (0.53) (Table 4).

**Table 4:** Means and Standard Errors (SE) for the Secondary Outcomes (Heat Pain Threshold [HPTh] and Heat Pain Tolerance [HPTo]) according to Group (a-tDCS + Go/no-Go Task *vs.* s-tDCS + Go/no-Go Task) (n = 35).

Pain measures	Sham tDCS M (SE)	Active tDCS M (SE)	Mean Difference (95% Confidence interval)	SMD	P value^a^
HPTh (°C)	38.88 (2.16)	40.81 (2.86)	1.93 (0.7 to 2.5)	0.89	0.03
HPTo (°C)	42.26 (2.86)	43.78 (2.21)	1.52 (0.12 to 2.91)	0.53	0.03

^a^Values based on a mixed ANOVA model. Significance level was P < 0.05.

### Relationship of ANT scores with pain measures and Group

We applied additional analysis to understand the relationship between attentional and pain measures. HPTh was significantly correlated with Executive attention in the multivariate linear regression model (Wilks’ λ = 0.81, F = 7.79 P = 0.002, Partial η2 = 0.19). HPTh was inversely correlated with Executive, and the effect size was small (Cohen’s f2 = 0.11). Estimators of the association in the multivariate model are presented in Table 5. On the other hand, there was no correlation between HPTh and orienting. Although tDCS influences pain and attention in FM, when the effects of tDCS on Executive attention is controlled for pain (as indexed by HPTh), there is a small decrease in the coefficient of treatment effect. However, this coefficient remained significant, suggesting the effects are mostly independent.

**Table 5:** Multivariate Regression Model for the Association between ANT and Pain measures and Group (a-tDCS + Go/no-Go Task *vs.* s-tDCS + Go/no-Go Task) (n = 35).

Dependent Variable	Type III Sum of Squares	df	Mean Square		F	P	Partial η^2^
ANT Orienting	736.4	2	368.2		0.36	0.70	0.01
ANT Executive	53545.6	2	26772.8		9.23	<0.01	0.23
		**Β**	**SEM**		**t**	**P**	**Partial η** ^**2**^
**ANT Orienting**							
Intercept		105.42	52.13		2.02	0.04	0.06
s-tDCS		−1.28	8.10		−0.15	0.87	<0.01
a-tDCS^a^		.	.		.	.	.
HPTh (°C)		−1.09	1.29		−0.84	0.40	0.03
s-tDCS*HPTh *vs* a-tDCS*HPTh		4.26	4.98		0.85	0.39	0.02
**ANT Executive**							
Intercept		375.56	87.61	4.29		<0.01	0.23
s-tDCS		−48.77	13.61	−3.58		<0.01	0.17
a-tDCS^a^		.	.	.		.	.
HPTh (°C)		−6.12	2.17	−2.82		0.01	0.11
s-tDCS*HPTh *vs* a-tDCS*HPTh		4.25	4.98	0.85		0.39	0.02

Notes: HPTh = Heat Pain Threshold; df = degrees of freedom; SEM = standard error of the mean; η^2^ = eta squared effect size index.

^a^Comparative group, to which values are referenced to.

### Effects of tDCS on the Go/No-go Task

To clarify training performance and possible effects of tDCS along the study, Table 6 presents means and SD according to the main scores of the Go/No-go Task. A mixed linear model analysis for each of the scores focusing on Group and Phase factors did not find any significant main effects of the conditions (Group or Phase), a no interaction. P values are presented in Table 6.

**Table 6:** Go No-go Task scores according to Group and Phase (n = 35).

Go/No-go Task scores	Phase 1 s-tDCS	Phase 1 a-tDCS	Phase 2 s-tDCS	Phase 2 a-tDCS	P value^a^
	M (SE)	M (SE)	M (SE)	M (SE)	
Proportion of correct Go trials (hits)	0.98 (0.01)	0.97 (0.01)	0.98 (<0.01)	0.97 (0.01)	0.744
Proportion of incorrect No-go trials (false alarms)	0.54 (0.03)	0.58 (0.03)	0.54 (0.04)	0.52 (0.04)	0.444
RTs of Go trials (hits)	438.1 (16.0)	417.9 (16.0)	402.3 (12.8)	403.6 (12.8)	0.462
RTs of incorrect No-go trials (false alarms)	424.8 (23.2)	425.0 (23.2)	408.9 (20.1)	383.5 (20.1)	0.558

Notes: s-tDCS = sham stimulation group; a-tDCS = active stimulation group; RTs = reaction time (in milliseconds).

^a^Refers to the F statistics for the interaction term between Group and Phase. No main effects were found for Group or Phase in any measure.

## Discussion

The present findings confirmed our hypotheses that active tDCS over the left DLPFC, as compared to sham, can produce significant changes in the performance of a task that is related to three attentional networks. Specifically, the data shows that a-tDCS, compared to sham, led to increased performance in the orienting and executive attention networks. There was, however, no difference due to tDCS on the alerting attentional network. Regarding our secondary outcome – the effects on pain - active tDCS increased HPT and pain tolerance, as compared to sham.

Previous studies have found that people with FM show reduced capacity to maintain the endogenous level of activation that is necessary to perform a task (reduced speed of processing) and impaired sustained attention, as compared to healthy controls^27^. In fact, people with FM, compared to healthy controls, show impaired performances in the ANT, namely impaired executive control, reduced general vigilance (slower reaction times) and greater alertness (more errors after a warning cue)^28^. In this study, data shows that anodal tDCS over the left DLPFC combined with a Go/No-go Task can significantly modulate the orienting (increased RTs) and executive (decreased RTs) attentional networks, as assessed by the ANT. Thus, it seems that anodal tDCS is able to improve the selection signal from sensory input (orienting), as well as the ability to monitor and solve interferences from different inputs and responses (executive), but without inducing any changes in the sensitivity to incoming stimuli (alerting)^29^.

Previous studies showed that subjects with FM have a reduction in their capacity to filter out distracting information^10^. This interference can impair the executive functioning and attention shifting in tasks containing a distraction as a competing source of information^19, 30^. So anodal tDCS seems to impact attention networks by a reduction of the cue’s predictive value, but also by improving conflict resolution among incongruent cues. Interestingly enough there was no effect on the alert attentional network. This may have happened due to the effect on the executive network, as it is thought that alerting network can have an inhibitory effect on the executive network, leading to faster responses to sensory input (infrequent stimulus) and preventing the system from focusing on irrelevant stimuli^31^.

Although the underlying mechanisms that contribute to cognitive dysfunctions in FM are not entirely explained, cognitive impairments seem to be associated with chronic stress related changes in the brain, namely hypocortisolism^32^, hippocampal dysfunction^33^ and alterations in prefrontal cortical morphology^34^. This maladaptive psychological mechanism of pain processing, as investigated in the present study, has been assumed to enhance vulnerability for developing or maintaining disability due to chronic pain. In fact, this finding is supported by previous longitudinal studies, which demonstrated that the connectivity between the default mode network and insular cortex is correlated with the pain level at the time of the scan^35, 36^. This hyper-connectivity of the insular cortex with regions involved in self-referential cognitions are thought to play a pivotal role on pain perception in fibromyalgia^35^. In the same line, two independent studies showed an association between pain relief following treatment with pregabalin and a decrease in the connectivity between the default mode network and insular cortex^36, 37^.

This finding is especially relevant because the DLPFC reflects cognitive-executive control^38^, but seems also to be able to influence the descendent pain modulation system^39^. Thus, the additive effect of a combined active tDCS with a task with inhibitory cues suggests that the activation of neural networks by non-pain related stimulus may be a top—down neuromodulatory approach with possible clinical impact for pain relief, as seen by the effect of anodal tDCS in the executive attentional network and pain relief. This finding is a potential insight to support the idea of using active tDCS combined with pharmacological intervention^40^ or conscious distraction^41^ in order to improve the treatment effects of fibromyalgia.

The active tDCS increases the HPT and pain tolerance (Table 5). This finding is supported by an extensive number of studies using tDCS to decrease pain^42–44^. Although the most common site of stimulation to treat pain has been the motor cortex (M1)^45^, the data from the present study suggests that anodal tDCS over the left DLPFC might affect sensory-discriminative pain processing, which in turn induces pain relief. This effect on pain relief has already been reported following tDCS over the left DLPFC^46^. Moreover, the involvement of the DLPFC on pain analgesia has been already well documented According to previous studies, the DLPFC is though to be involved in placebo analgesia in downstream circuits to the anterior insula, ACC, hypothalamus and the PAG^47, 48^. What is not known is how anodal tDCS over the Left DLPFC can induce pain relief. One hypothesis is that tDCS over the DLPFC increases connectivity across brain regions^49, 50^. For instance, tDCS over M1 may increase the activity of insula and thalamus^51, 52^. Moreover, anodal tDCS over M1 increases functional coupling between ipsilateral M1 and thalamus^53^. Thus, one hypothesis is that the analgesic effects observed after the DLPFC stimulation may be least partially attributable to an indirect inhibitory modulation of thalamic activity, that well result in the alleviation of the painful experience after stimulation of the DLPFC^46^.

Nonetheless, there are some potential limitations in the present design. A uniform dose of stimulation was used for all subjects, and this may not be the most efficient procedure^54^. Also, we included only females because they are more prone to activation upon negative emotional responses (i.e., stress, fear, and anxiety). Thus, in this context, the gender may be a significant confounding factor. Also, there is no control group. We chose a crossover design, because it will allow us to have subjects serving as their own controls, thus reducing variability when assessing outcomes related to behavior and physiological parameters^43^. And finally, although the present findings are important to understand the possible neurobiological mechanisms underlying the effects of tDCS over DLPFC in combination combined with go/no-go task in attention networks and pain modulation, they do not provide enough evidence in order to guide decision making in clinical settings. However, future studies should test the addictive effects of repetitive sessions of tDCS, especially in these types of chronic conditions. For instance, another study using tDCS with fibromyalgia patients showed that the median number of sessions required to induce a clinically meaningful effect was 15^55^.

## Conclusion

Overall, our results highlight two important conclusions. First, one session of anodal tDCS over the left DLPFC has a modulatory effect on the orienting and executive attentional networks, as assessed by the performance of the ANT. The secondary effect in pain could reflect an active control of pain perception in a top-down manner. Also, these findings suggest that the effect of tDCS on pain and attention may be a major target for neurostimulation therapies in addition to or in combination with the primary motor cortex for subjects who do not respond or are more refractory to neurostimulation therapies.

## Methods

### Design overview, settings and participants

The methods and results sections are reported according to the CONSORT guidelines. All subjects provided written informed consent before participating in this randomized, crossover blind, clinical trial with allocation ratio of 1:1. The study followed the guidelines and regulations for clinical research and was approved by the Research Ethics Committee at the Hospital de Clínicas de Porto Alegre (HCPA) (Institutional Review Board IRB 140231).The current controlled trial is registered at ClinicalTrials.gov under number NCT02454218 (First received: January 19, 2015).

We recruited 40 adult from 18 to 65 years-old female outpatients of the HCPA and via advertisement. Sample size was calculated based on previous findings, with 0.8 effect size (Cohen’s D) magnitude (SD = 0.6), alpha level of 0.01 and 80% power. FM was diagnosed according to American College of Rheumatology criteria^56^. Subjects were required to have a score of at least 50 mm on the 0–100 mm visual analogue scale for pain (VAS, which 0 means “no pain” and 100 means “worst possible pain”) during most of the days over the last three months^57^. Subjects were allowed to remain on analgesic medications, including drugs for which they were refractory, and these medications could not be adjusted during the study. Major depressive disorder was accepted as secondary to FM. Subjects with history of substance abuse or evidence of other pain-related disorder were excluded. Females pregnant, in breast-feeding, or with a history of neurologic or oncologic disease, ischemic heart disease, kidney or hepatic insufficiency were also excluded.

### Intervention: Online combined tDCS stimulation and Go/No-go Task

tDCS was applied by a Research Limited stimulator (TCT, Hong Kong, China) using 35 cm^2^ saline-soaked electrode sponges. Each tDCS session started with 3 minutes^46^ of stimulation applied to each subject prior to execution of the computerized Go/No-Go task^58^. This interval was used because it has been reported to be the minimum amount of time required for tDCS to elicit after-effects^49^. Subjects were randomly assigned to receive two sessions of tDCS (anodal a-tDCS and sham) over the left DLPFC. The active electrode (anode, 35 cm^2^) was placed over the left DLPFC [F3, 10–20 system], and the return electrode (cathode, 35 cm^2^) was placed on the contralateral supraorbital area (FP2 site). tDCS was applied with 1 mA intensity, for a total of 20 minutes, with a 30-s ramp up and down. Sham started with a 15-s ramp up and immediately 15 s ramp down), with no current flowing after this.

All tasks were presented using E-Prime version 2.0 SP1 software (Psychology Software tools, Sharpsburg PA, US). Stimuli were presented on the center of screen with approximately 2.0° of visual angle (viewing distance approximately 60 cm). The Go/No-go Task is a simple choice reaction paradigm that requires response inhibition when one of two stimuli is presented (go versus no-go). On the center of the screen, subjects were shown a fixation cross (1000 ms) followed by a go letter (e.g., “L”, “Q”, “B”, etc.) or a no-go letter (e.g. “X”) for 500 ms each and were instructed to press the “space” key as fast as possible for the go letters. They were required to not press any key for the no-go letters. There were three blocks, each with a different no-go letter (“H”, “X” and “K”) to avoid learning (no-go letters were never presented as a go letter), with a total of 339 go trials and 78 no-go trials. Total task time was 17 minutes.

### Randomization

We used a simple randomization method via Randomization.com, which relies on independent and equal probabilities to receive each intervention for each subject. Although it is the most basic approach, it preserves unpredictability of the allocation. The software generated a random number, for which one of the interventions was randomly allocated. In this way, it was guaranteed that each subject had the same chance of receiving one of the interventions (active or sham) first.

### Blinding

Subjects were instructed to discuss all aspects related to their tDCS intervention only with the person responsible for the tDCS application (rather than the person responsible for the assessments)^44^. Allocation concealment was assured by intervention being assigned only after enrollment. Furthermore, to assess whether blinding was effective, at the end of the experiment subjects were asked to guess whether they had received a-tDCS or sham and to rate their confidence level using a 5-point Likert scale (from no confidence at all to completely confident).

### Baseline instruments and assessments

All psychological tests used have been validated for the Brazilian population. Two independent medical examiners, who were blinded to the group assignments were trained to conduct the psychological tests and administer the pain scales. At the baseline, the instruments used were: Pittsburgh Sleep Quality Index^59^ to assess the sleep quality; Beck Depression Inventory-II (BDI-II^60^), for the assessment of depressive symptoms; Mini-International Neuropsychiatric Interview (M.I.N.I.^61^) to detect psychiatric disorders; The Brazilian validated version of the Fibromyalgia Impact Questionnaire (FIQ^62^), to assess quality of life of FM patients; and the Brazilian Portuguese version of the Pain Catastrophizing Scale (B-PCS^63^), for the catastrophic thinking. Medical comorbidities and demographic data were assessed using a standardized questionnaire. To evaluate the safety of tDCS, we used the Systematic Assessment for Treatment with tDCS questionnaire based on previously reported adverse events^64^.

### Outcomes

The primary outcome was the effects of the intervention on the performance of the ANT, based on the scores of Alerting, Orienting, and Executive attention. Secondary outcomes were the temperature reported for the heat pain threshold (HPTh) and heat pain tolerance (HPTo).

#### Attention Network Test (primary outcomes)

**Figure 2:**
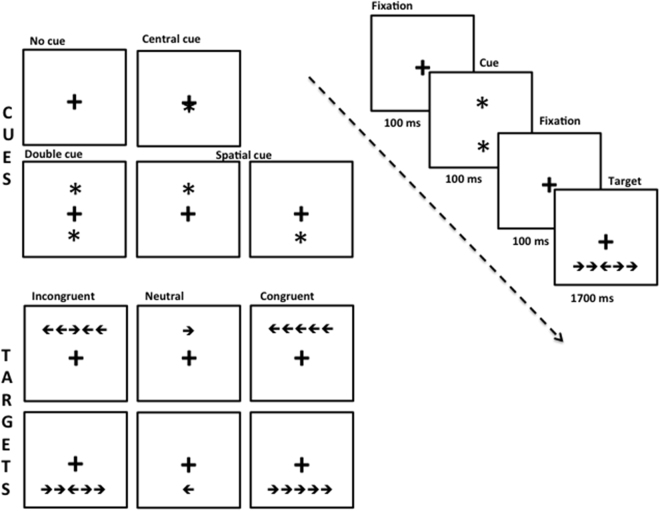
Attention network test (ANT); cues: no cue, central cue, double cue and spatial cues; and targets: Incongruent, neutral and congruent and an example of the procedure of ANT. Left column: sequence of events per trial of the ANT; right column: possible stimuli associated with each event. Except for the spatial and invalid spatial cue (80% vs. 20% probability, respectively), all cue and flanker constellations were equally probable and appeared up or down of the fixation cross. The target and flanker remained visible on the screen until the patients respond, but for no longer than 1700 ms. While trial duration was fixed to 4000 ms, a temporal jitter was introduced by a variable delay of the cue onset (200, 300, and 400 ms after trial onset) to reduce expectancies.

The ANT^65^ was used to assess the attention networks performance. A target (i.e. arrows) pointing left or right appeared on the center of the screen for 1700 ms right after a cue, which lasted for 100 ms (i.e. asterisk). The ANT is a combination of a cued reaction time task with the flanker test. Therefore, the test had four Cue (warning) conditions (no cue, central cue, double cue and spatial cue) and the three Target (flanker) conditions (congruent, incongruent and neutral); see Fig. 2 for a detailed view.

All combinations of conditions (cue and target) were randomly presented in one block of 96 test trials. Twenty-four practice trials were performed before the test trials. Subjects were asked to identify the direction to which the center arrow pointed as soon as possible. Based on cue and target conditions, three main indices were calculated. The Alerting attention was calculated by subtracting the mean response time (RT) of the double-cue conditions from the mean RT of the no-cue conditions. The Orienting attention was calculated by subtracting the mean RT of the spatial cue conditions from the mean RT of the center cue. For these two measures, the higher the score, the better the participant’s attentional processing. Finally, the Executive (conflict) attention was calculated by subtracting the mean RT of all congruent flanking conditions from the mean RT of incongruent conditions^65^. For this measure, the lower the score, the better the participant dealt with interference. Scores over 2 standard deviations from the mean were removed (which represents less than 5% of the total number of scores).

#### Pain measures (secondary outcomes)

Pain was assessed using a computer Peltier-based device thermode (30 × 30 mm)^66^. The thermode was attached to the skin on the ventral aspect of the mid-forearm, with temperature increasing 1 °C/s, from 32 °C to a maximum of 52 °C (for safety reasons, after which the device cooled down). The heat primarily stimulates C-nociceptive afferent pathways^67^. Using the methods of limits, the participants were asked to press a button with the thumb as soon as they feel the stimulus as painful. Three assessments were performed with an interstimuli interval of 40 s, with position of the thermode being slightly altered (approx. 30 mm) to avoid sensitization or response suppression. The average of those assessments is the Heat Pain Threshold (HPTh). Heat Pain Tolerance (HPTo) temperature was determined by asking subjects to press a button as soon as the sensation of pain had reached the maximum they could tolerate. In case 52 °C was the subject’s maximum temperature, the HPTo was considered unknown.

### General Procedure

**Figure 3:**
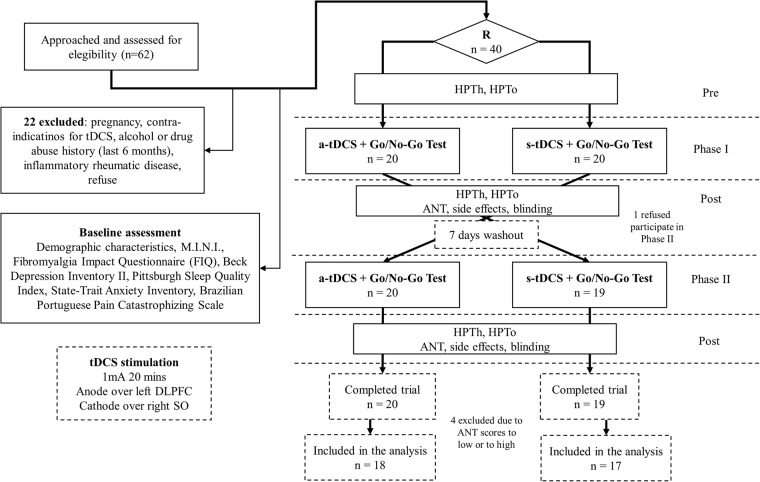
Flowchart showing recruitment and progress through the study.

Participants initially volunteered by signing the consent form. Following this, they responded to the baseline assessments and were checked for necessary exclusion criteria. Then, they were randomly allocated to one of the experimental groups, either receiving sham stimulation first (Sham-Active Group) or active stimulation first (Active-Sham Group). Measures of attention through the ANT were obtained after each phase of tDCS, with a 1-week interval between each phase. Blinding was also incorporated and side effects following tDCS were recorded. Heat pain measures were assessed before and after each phase, although only after measures were analyzed here. Data was collected in acclimatized rooms of the Clinical Research Center in the hospital. Figure 3 presents the flowchart of the study.
